# Effect of nitrogen atomic percentage on N^+^-bombarded MWCNTs in cytocompatibility and hemocompatibility

**DOI:** 10.1186/1556-276X-9-142

**Published:** 2014-03-25

**Authors:** Mengli Zhao, Ye Cao, Xiaoqi Liu, Jianhua Deng, Dejun Li, Hanqing Gu

**Affiliations:** 1 College of Physics and Materials Science, Tianjin Normal University, Tianjin 300387, China; 2 Tianjin Institute of Urological Surgery, Tianjin Medical University, Tianjin 300070, China; 3 School of Medicine, Ninth People's Hospital, Shanghai Jiao Tong University, Shanghai 200011, China

**Keywords:** N^+^-bombarded multi-walled carbon nanotubes, Nitrogen atomic percentage, Ion beam-assisted deposition, Cytocompatibility, Hemocompatibility

## Abstract

N^+^-bombarded multi-walled carbon nanotubes (N^+^-bombarded MWCNTs), with different nitrogen atomic percentages, were achieved by different N ion beam currents using ion beam-assisted deposition (IBAD) on MWCNTs synthesized by chemical vapor deposition (CVD). Characterizations of N^+^-bombarded MWCNTs were evaluated by X-ray photoelectron spectroscopy (XPS), transmission electron microscopy (TEM), Raman spectroscopy, and contact angle. For comparison, the *in vitro* cytocompatibility of the N^+^-bombarded MWCNTs with different N atomic percentages was assessed by cellular adhesion investigation using human endothelial cells (EAHY926) and mouse fibroblast cells (L929), respectively. The results showed that the presence of nitrogen in MWCNTs accelerated cell growth and proliferation of cell culture. The higher nitrogen content of N^+^-bombarded MWCNTs, the better cytocompatibility. In addition, N^+^-bombarded MWCNTs with higher N atomic percentage displayed lower platelet adhesion rate. No hemolysis can be observed on the surfaces. These results proved that higher N atomic percentage led N^+^-bombarded MWCNTs to better hemocompatibility.

## Background

The last decade has seen a great deal of activity in the use of carbon nanotubes (CNTs) to augment the properties of a variety of materials, including biomaterials [1]. The advantage of carbon nanotubes in biomedicine is their stable conductivity in aqueous physiological environment, thus making them attractive for cellular stimulation [2]. And, the weakness of raw CNTs is their super-hydrophobicity. They can easily aggregate in aqueous media as well as in organic solvents, which strictly restricts their application in biomedical fields because a hydrophilic interface is in favor of enhancing bioactivity [3]. So, in recent years, the enormous progress in nanotechnology and material sciences had stimulated the development and production of engineered carbon nanotubes [4-9]. And, numerous studies in biomaterial development indicated the functionalized water-soluble CNTs to improve cell attachment and growth [5-9]. In our previous work [10], the improved hemocompatibility and cytocompatibility were also observed in N-doped MWCNTs when compared with pristine MWCNTs using chemical vapor deposition (CVD) method. Recently, many studies on the functionalization of MWCNTs have been reported. Chemical grafting is the main method for CNT functionalization. In previous works, we also synthesized MWCNTs containing N, carboxyl, and hydroxyl groups using CVD and compared the biocompatibility of MWCNTs with and without functional groups [10-12]. A significant improvement in cell and blood behaviors was observed in MWCNTs containing functional groups compared with pure MWCNTs. However, few reports are found to achieve MWCNT functionalization using the ion beam bombardment or ion implantation technique. The advantages of the physical method are its simplicity, small amounts of impurities, and high content of active groups on the surface of MWCNTs. Differing from the traditional chemical grafting, the ion implantation technique was also used to introduce NH_2_ and COOH groups onto MWCNTs, and graphene which was found to result in favorable effects on their biocompatibility in our previous works [13-16].

To differ from traditional chemical grafting and ion implantation, in this paper, lower-energy N ion beam bombardment method was used to introduce N ions to MWCNTs. Compared with ion implantation, the advantages of low-energy ion beam bombardment are its shallow injection depth and high content of active nitrogen on the surface of MWCNTs. The interaction between cell and substrates primarily occurred on the shallow surface of modified MWCNTs. The larger number of active nitrogen on the surface of MWCNTs which interacted with cells *in vitro* could increase the number of sites for cell growth. Thus, the modified MWCNT surface should have better bioactivity and biocompatibility.

Due to length limitation, the comparison between pure and N^+^-bombarded MWCNTs in cytocompatibility and hemocompatibility will be submitted to other journals. This work only focused on the relationships between cell and blood behaviors and N atomic percentages of laboratory-made MWCNTs bombarded at different N^+^ beam currents (5, 10, and 15 mA), which were evaluated by cell adhesion, hemolysis, and platelet adsorption.

## Methods

### Synthesis

MWCNTs were prepared using CVD system and then sprayed onto SiO_2_ substrates with air brush pistol. The detailed process of sample preparation can be found in our previous work [17,18].

An ion beam-assisted deposition (IBAD) system (FJL560C12, SKY Technology Development Co., Ltd., China) was used to prepare N^+^-bombarded MWCNTs. This system has two ion sources, one water-cooled sample holder and one water-cooled target holder. In this processing, the chamber was evacuated to a base pressure lower than 3.0 × 10^-4^ Pa prior to N ion bombardment. Then, the high-purity N_2_ gas was introduced into low-energy ion source which could perform N ion bombardment to MWCNTs at desired ion bombarding parameters through computer controlling. N ion beams at ion beam currents of 5, 10, and 15 mA and a constant bombarding energy of 200 eV were respectively accelerated to bombard MWCNTs for 30 min to get three N atomic percentages of N^+^-bombarded MWCNT samples. The working gas pressure was 1.2 × 10^-2^ Pa.

### Contact angle, XPS, SEM, TEM, and Raman analysis

Water contact angles were measured using a face contact angle meter (CAM KSV021733, Nunc, Finland). The detailed measurement process can be found in our previous work [17-19]. Characterization by X-ray photoelectron spectroscopy (XPS) (PHI5000 VersaProbe system, Physical Electronics, Chanhassen, MN, USA) was used to prove the existence of the main functional groups in the three samples. The morphology of N^+^-bombarded MWCNTs was examined with a field emission scanning electron microscope (FESEM; 18SI, FEI, Hillsboro, OR, USA) operated at 10.0 kV and a field emission scanning electron microscope (SU8020, HITACHI, Tokyo, Japan) operated at 1.0 kV. The detailed morphologies and chemical bonding states of the samples were characterized using a JOEL JEM 2100 transmission electron microscope (TEM; Tokyo, Japan) and Renishaw micro-Raman 2000 system (Wotton-under-Edge, UK) and a 514-nm laser line excitation.

### Cell adhesion assays

The human endothelial cell line EAHY926 and mouse fibroblast cells (L929) were used to investigate the cytocompatibility of N^+^-bombarded MWCNTs. The processes of cell culture and cell vaccination can be found in our previous work [13-16]. Endothelial cells were harvested from the cultures and replaced into 24-well plate (5 × 10^4^ cells/ml) in four groups (three kinds of N^+^-bombarded MWCNTs and blank control group). The inoculum density of fibroblast cells is 2.5 × 10^4^ cells/ml. After 1 to 7 days in an incubator (culture intervals of 0.5, 1, 2, 3, 5, and 7 days), the medium was removed, and the cell monolayer was washed several times with PBS and then isolated by trypsin for enumeration.

Immunofluorescence staining was done as described with mouse monoclonal anti-α-tubulin (clone B-5-1-2, 1:1,000 dilution; Sigma, St. Louis, MO, USA), followed by 1:200 dilution of various fluorochrome-conjugated secondary antibodies. Finally, DNA was stained with DAPI (1 μg/ml) for 5 min. For immunostaining, mouse fibroblast cells were grown on three kinds of N^+^-bombarded MWCNTs at 2.5 × 10^4^ cells/ml for 24 h. Confocal scanning laser microscopy (CSLM) (Nikon Eclipse 90, Shinjuku, Tokyo, Japan) was employed to observe cell morphology and stretching on the three samples. The scanning electron microscope (SEM) (FEI QUANTA 200) was employed to observe endothelial cells' and mouse fibroblast cells' morphology and stretching on three materials.

### Hematotoxicity analysis

Platelet adhesion test was conducted to evaluate the surface thrombogenicity of the materials *in vitro*. Blood taken from a healthy rabbit with potassium oxalate as the anticoagulant was centrifuged about 15 min and converted to platelet-rich plasma (PRP). All the N^+^-bombarded MWCNTs and reference groups were cleaned and then incubated in human PRP for 30 min at 37°C. The detailed process can be found in our previous work [17,18]. Since methylsilicone oil has excellent anticoagulant activity, but quartz causes coagulation, we chose two quartz glasses with and without methylsilicone oil which had the same size with N^+^-bombarded MWCNT samples as positive and negative reference groups, respectively. The platelet adhesion rate of a material can be represented as follows:

$$\text{Platelet}\;\text{adherent}\;\text{rate}\;\left(\%\right)=\frac{A-B}{A}\times 100\%,$$

where *A* is the total number of platelets and *B* is the number of platelets remaining in the blood after the platelet adhesion test [20]. The morphology of adherent platelets was assessed using SEM.

Anticoagulant blood solution was obtained by adding normal saline to anticoagulant blood which was prepared from healthy rabbit blood plus 2% potassium oxalate. The samples were placed in each Erlenmeyer flask and added with 5 ml normal saline. The same numbers of Erlenmeyer flasks with either 5 ml normal saline or distilled water were used as negative and positive control groups, respectively. The detailed process can be found in our previous work [10,17,18].

## Results and discussions

**Figure 1:**
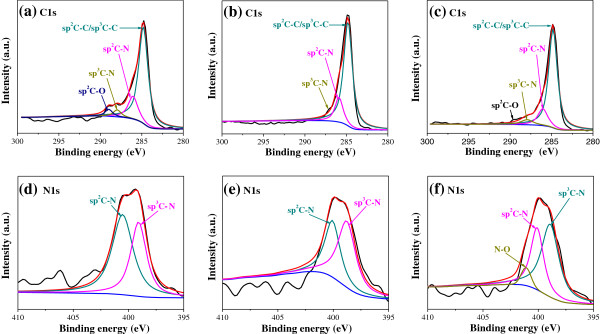
**XPS spectra analysis of C1s and N1s for N**^**+**^**-bombarded MWCNTs.** Nitrogen contents are **(a, d)** 7.81%, **(b, e)** 8.67%, and **(c, f)** 9.28%.

From the XPS analyses, the nitrogen concentration of three N^+^-bombarded MWCNTs is 7.81%, 8.67%, and 9.28% at the corresponding bombarding beam current density of 10, 15, 5 mA, respectively. The result shows that the nitrogen concentration does not increase as the bombarding beam current density increases. We suppose that the binding between N^+^ and MWCNTs is not stable. The previously formed groups are destroyed by N^+^ ions as the beam current density increases. Figure 1 shows the peak position and area of the analyses of C1s and N1s. The main peak of the C1s is *sp*^2^ and *sp*^3^ carbon atoms at 284.6 eV [21,22]. Furthermore, C1s peaks of N^+^-bombarded MWCNTs also revealed *sp*^2^ and *sp*^3^ C-N bondings at 285.5 and 287.4 eV, respectively [22]. The N1s spectra are decomposed into two peaks which are located at 398.5 and 400.5 eV, respectively, being attributed to *sp*^3^ and *sp*^2^ C-N bondings [23] correspondingly. From the data, it indicates clearly that with the increase of nitrogen concentration, the ratio of the *sp*^2^ C-N bond decreases, and the *sp*^3^ C-N bond increases while the unsaturated degree of the N bond increases.

**Figure 2:**
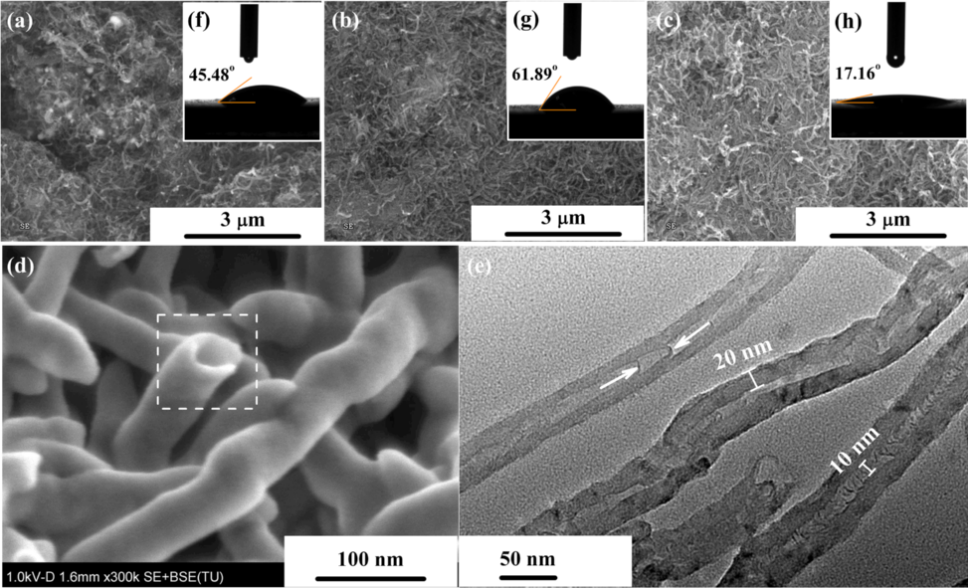
**SEM images of N**^**+**^**-bombarded MWCNTs.** Nitrogen contents are **(a)** 7.81%, **(b)** 8.67%, and **(c, d)** 9.28%. **(e)** TEM image of N^+^-bombarded MWCNTs with nitrogen content of 9.28%. The insets **(f, g, h)** are their contact angle images, respectively.

Figure 2a,b,c presents the SEM images of the N^+^-bombarded MWCNTs at ion beam currents of 5, 10, and 15 mA, respectively. From Figure 2a, it can be seen that N^+^-bombarded MWCNTs completely covers the substrate at high density, and the surface is rough. As ion beam current increases, more fractured N^+^-bombarded MWCNTs fill the gaps between them, resulting in smoother surface (Figure 2b,c). To investigate the detail morphology of N^+^-bombarded MWCNTs, SEM with high magnification and TEM characterizations are performed, as shown in Figure 2d,e. N^+^-bombarded MWCNTs' tube structure is proved (shown by white rectangle in Figure 2d). From Figure 2e, the graphite layers of MWCNTs are parallel to each other. The N^+^-bombarded MWCNTs used in these studies have a diameter distribution of 40 to 60 nm and few microns in length. And, the wall thicknesses are around 20 nm. The N^+^-bombarded MWCNTs show bamboo-like structure enriched by nitrogen element [24-26], in which interlinks are observed within the tubes (shown by white arrow in Figure 2e). In addition, the tube wall of the N^+^-bombarded MWCNTs has irregularities, indicating the deformation of their structure. The structural change of the N^+^-bombarded MWCNTs is probably caused by the introduction of nitrogen element.

Wettability, evaluated through the measurement of the contact angle of a liquid on a surface, is a sensitive way to detect surface modifications [27]. Furthermore, it is a measurement of the hydrophilic/hydrophobic character of a material, a relevant property regarding biocompatibility, since it has a major influence on protein adsorption and interaction with cells [28]. In this work, the wettability of the three samples was evaluated by water contact angle measurements, as shown in Figure 2f,g,h. The values of N^+^-bombarded MWCNTs at nitrogen concentrations of 7.81%, 8.67%, and 9.28% are 61.89°, 17.16°, and 45.48°, respectively. It is worth noting that the increase of contact angle is not related to the increase of nitrogen concentration and ion beam current. The results show a slight decrease in contact angle with the decrease of the *sp*^2^ C-O content.

**Figure 3:**
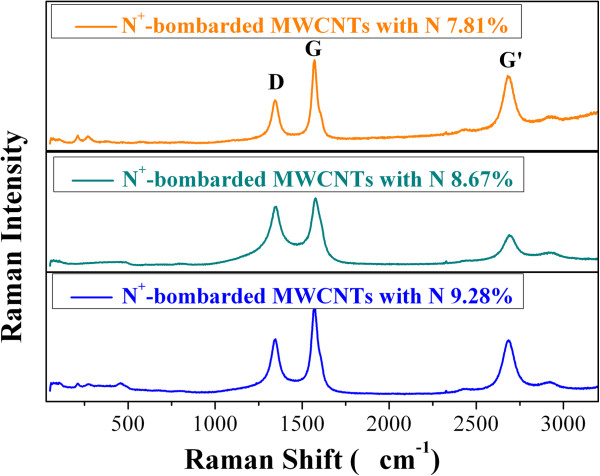
**Raman spectra for N**^**+**^**-bombarded MWCNTs with three N atomic percentages.**

The Raman spectra of N^+^-bombarded MWCNTs at three N atomic percentages are shown in Figure 3. As can be observed, the samples show the typical D-mode (1,350 cm^-1^) and G-mode (1,590 cm^-1^) vibration bands and overtone of the D-mode (G′ 2,680 cm^-1^). A major effect of N introduction is increase clustering of the *sp*^2^ phase, which is indicated by the D peak [29]. In this study, we refer to *I*(D)/*I*(G) as the ratio of peak heights. In amorphous carbons, the development of a D peak indicates ordering [30]. So, it is noticeable that the ratio of *I*(D)/*I*(G) for N^+^-bombarded MWCNTs with N 8.67% atomic percentage is higher than those of the other samples, implying that nanotube destruction and creation of amorphous carbon impurities are introduced in the N ion bombardment.

**Figure 4:**
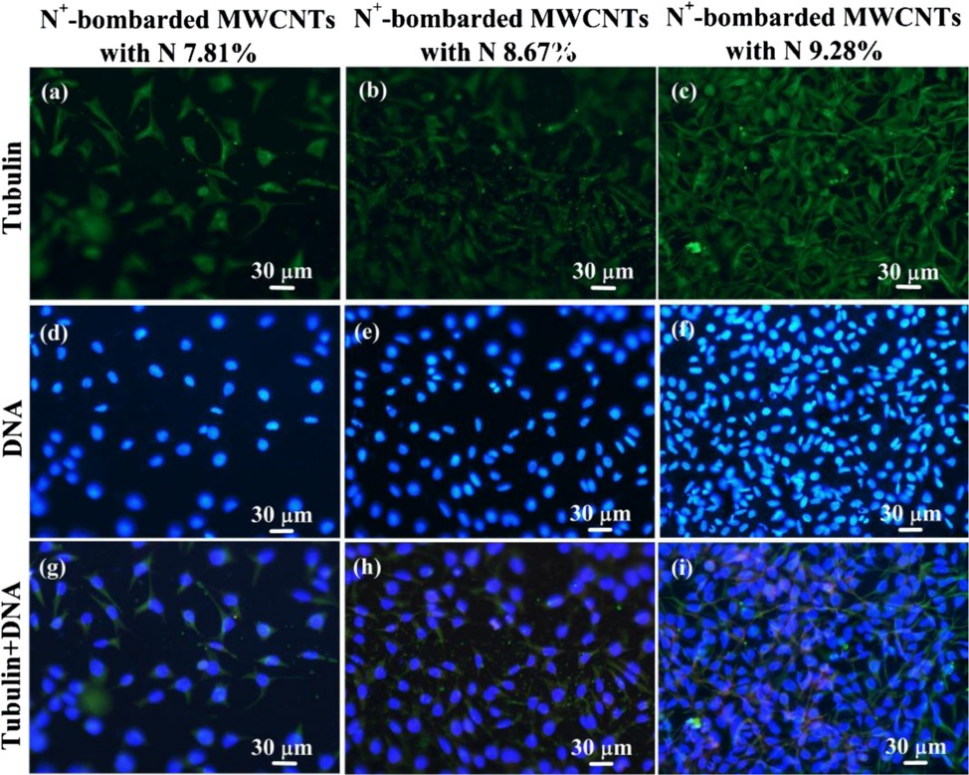
**CSLM images of mouse fibroblast cells fixed on N**^**+**^**-bombarded MWCNTs.** Nitrogen contents are **(a, d, g)** 7.81%, **(b, e, h)** 8.67%, and **(c, f, i)** 9.28%.

Using immunofluorescence techniques, microtubules are stained, which are the main components of the cytoskeleton (shown in Figure 4a,b,c). Meanwhile, the nuclear DNA was stained with a different fluorescent dye (Figure 4d,e,f) and then the two photographs taken by CSLM in the same viewing field were combined, with same exposure times, as shown in Figure 4g,h,i. The CSLM images show the morphology of mouse fibroblast cells fixed on the surface of three samples after an incubation of 1 day. It can be seen from Figure 4a,b,c that typical triangular cells adhere to the surface of all the samples. The cells spread flat, and there are pseudopodia and microvilli on the cell surface. In addition, with the increasing nitrogen concentrations, the cell numbers on the materials become more and more. The nuclear DNA shows good cell viability on the surfaces of N^+^-bombarded MWCNTs, as shown in Figure 4d,e,f. And, it is clear that some DNA edges are smooth and blurred, while others are crisp and clear. This means that the mouse fibroblast cells grow on the three-dimensional configuration of N^+^-bombarded MWCNT samples. This structure offers a larger substrate area for cell growth and proliferation. Taken together, these results indicate that nonspecific binding between nitrogen in the N^+^-bombarded MWCNTs and cell surface proteins enhances cell adhesion and growth on the N^+^-bombarded MWCNTs.

**Figure 5:**
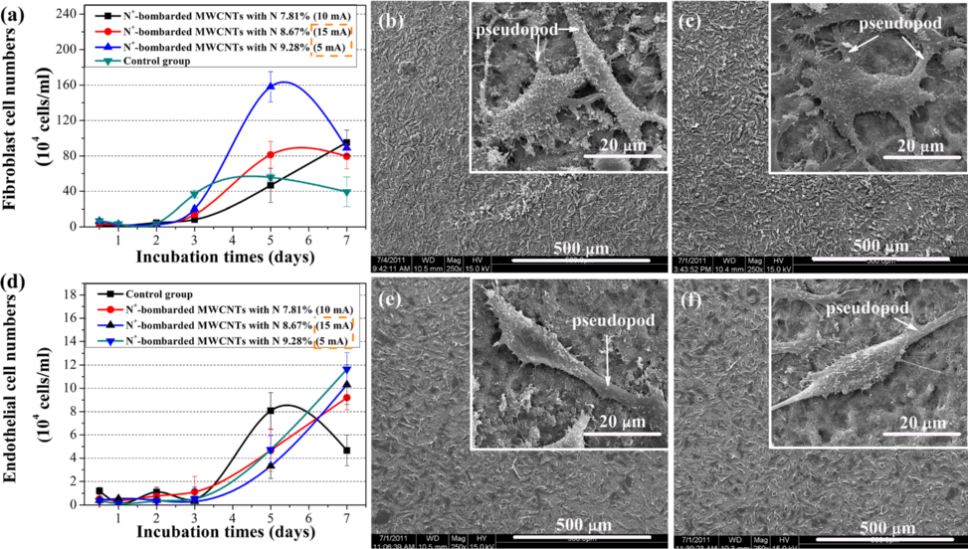
**Direct contact measurements and calculations of cell viability. (a)** L929 mouse fibroblast cell numbers on the surfaces of different materials vs. incubation time; SEM images of L929 mouse fibroblast cells fixed on the surfaces of N^+^-bombarded MWCNTs with nitrogen contents of **(b)** 8.67% and **(c)** 9.28%. **(d)** EAHY926 endothelial cell numbers on the surfaces of different materials vs. incubation time; SEM images of EAHY926 endothelial cells fixed on the surfaces of N^+^-bombarded MWCNTs with nitrogen contents of **(e)** 8.67% and **(f)** 9.28%.

In order to further verify the relationship between the nitrogen concentration and the cell adhesion, we choose mouse fibroblast cells and human endothelial cells for direct contact measurements and calculations of cell viability at 0.5, 1, 2, 3, 5, and 7 days through a biological inversion microscope, as shown in Figure 5a,d. Each value in these figures represents the mean ± SD for five measurements. And, each experiment is performed three times. It can be seen from the two figures that the cell concentrations of N^+^-bombarded MWCNTs and control group increase gradually from 1 to 5 days, and no dead cells are observed under the microscope in all samples. The cell adhesion numbers on N^+^-bombarded MWCNTs increase with increasing nitrogen concentration. After 5 days, the mouse fibroblast cell numbers of N^+^-bombarded MWCNTs reduce gradually as the concentration of the control group reduced (Figure 5a).

Endothelial cells have been shown to be more sensitive than mouse fibroblast cells to the same sample. The numbers of endothelial cells on N^+^-bombarded MWCNTs still increase rapidly after the 5-day incubation. And, it far exceeds the control group on the seventh day (Figure 5d). The highest nitrogen concentration displays the highest cell numbers. Thus, the high nitrogen concentration stimulates cell growth and proliferation of cell culture, revealing superior cytocompatibility.

Figure 5b,c,d,e,f shows clearly the difference at the amount and morphology of the adhered cells on N^+^-bombarded MWCNTs with N 8.61% and 9.28%. As we can see from the SEM images with low magnification, the cell concentration with N 8.67% (Figure 5b,e) is significantly less than that with N 9.28% (Figure 5c,f), which is consistent with the results given by Figure 4 and Figure 5a,d. And, the adhered cells all spread flat with richer pseudopod and microvilli, as shown at a high magnification. These results add to growing evidence that the increase of nitrogen content promoted cell adherence and growth.

The ability of substrates to promote adhesion of cells depends on how well they adsorb proteins from the culture medium that interact with receptors on the cell surface [31]. Adsorption of proteins in an active conformation, in turn, is likely to be affected by the functional groups of the substrate. All proteins have NH_2_ and COOH groups at the ends, where the NH tends to be positively charged and the COOH negatively charged [32]. Thus, a surface with an organized arrangement of functional groups can act as a site for cell growth. The formation of functional *sp*^2^ C-N and *sp*^3^ C-N bonds on the N^+^-bombarded MWCNTs by N ion beam bombardment induces polarization at the surface due to the difference in electronegativity between carbon and nitrogen [33]. In addition, from the XPS results (Figure 1d,e,f), it is clear that with the increase of nitrogen concentration, the ratio of the *sp*^2^ C-N bond decreases and the *sp*^3^ C-N bond increases while the unsaturated degree of the N bond increases. Therefore, the number of protein attached on the material's surface increases with increasing unsaturated degree of the N bond, and adhesion of cells are promoted.

**Figure 6:**
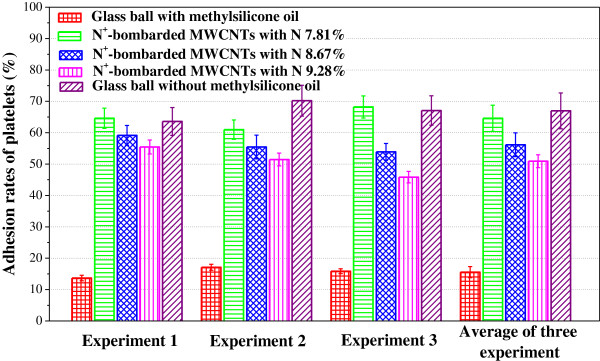
Platelet adhesion rates on the different materials.

**Figure 7:**
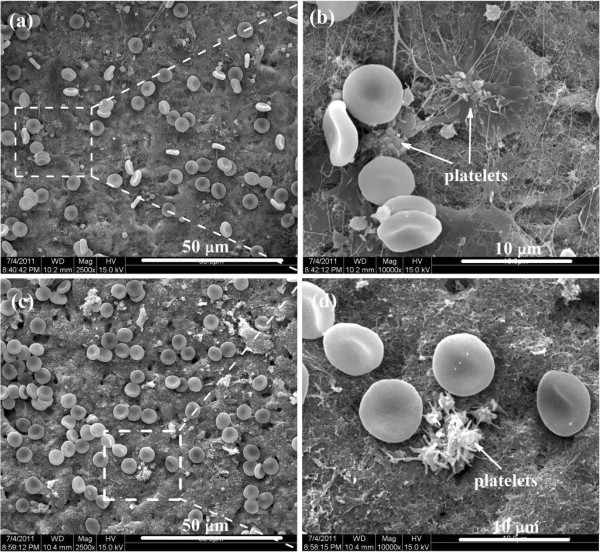
**SEM images of platelet adhesion testing for N**^**+**^**-bombarded MWCNTs.** Nitrogen contents are **(a, b)** 8.67% and **(c, d)** 9.28%.

Blood platelets are anucleated cells that originate from bone marrow megakaryocytes and circulate in the blood as sentinels for vascular integrity [34]. Platelets play a vital role in hemostasis; however, derangement of their functions can lead to thrombosis, which is a leading cause of death and disability in the developed world [35]. Figure 6 displays the statistical results of the platelets adhered on the surfaces of three N^+^-bombarded MWCNTs with different nitrogen content and the glass with and without methylsilicone oil. Each value represents the mean ± SD for five measurements. And, each experiment is performed three times. From the average platelet adhesion rates, it is observed that the number of adherent platelets decreases with increasing nitrogen concentration. In addition, as shown in Figure 7c,d, the platelets show less pseudopodium as demonstrated by the isolated and nearly round state when the nitrogen concentration is higher. The morphology of the red blood cell (RBC) on N^+^-bombarded MWCNTs is perfect round. It is demonstrated that higher nitrogen concentration is contributive to the improvement of hemocompatibility.

The morphological change of the adherent platelets is a common qualitative criterion to assess activation of adherent platelet on the materials' surface. Baurschmidt [36] reported that the formation of thrombus on the biomaterial surface is correlated with charge transferring from fibrinogen to the material surface. Fibrinogen can transform to fibrin monomer and fibrinopeptides when it losses charge. The crosslink of fibrin monomer causes an irreversible thrombus. Thus, the suitable density of charge will promote the hemocompatibility [37,38]. A suitable ratio of *sp*^3^ C-N to *sp*^2^ C-N can provide the optimum density of charge to promote hemocompatibility. The possible reason for the decrease of platelet adhesion rates is the significant change in the electronic characteristics due to the increase of *sp*^3^ C-N bond.

The hemolysis ratio was calculated by the formula $\mathrm{Hemolytic}\;\mathrm{rate}\;\left(\%\right)=\frac{A-B}{C-B}\times 100\%$, where *A*, *B*, and *C* are the absorbance values of the specimens, negative control group (physiological salt water), and the positive control group (H_2_O), respectively [17,18]. The average OD values of the N^+^-bombarded MWCNTs with 7.81%, 8.67%, and 9.28% are 0.027, 0.029, and 0.026, respectively. The hemolytic rates of all the N^+^-bombarded MWCNTs are all 0%. According to the YY/T0127.1 standard, a hemolytic rate below 5% is acceptable [38-40]. These results indicate that the three materials all have good hemocompatibility.

## Conclusions

In this paper, the cytocompatibility and hemocompatibility of the N^+^-bombarded MWCNTs with three N atomic percentages are investigated and compared. The cell adhesion assays indicate clearly that with the increase of nitrogen concentration, the ratio of the *sp*^2^ C-N bond decreases and the *sp*^3^ C-N bond increases while the unsaturated degree of the N bond increases. It may increase the number of protein which attached on the material's surface; so, the adhesion of cells is promoted. Thus, the cytocompatibility of N^+^-bombarded MWCNTs are promoted with the increase of nitrogen concentration. The blood experiments also show that N^+^-bombarded MWCNTs with higher nitrogen content displayed lower platelet adhesion rates and lower hemolytic rate values. In conclusion, bombarding N ions into MWCNTs by IBAD is a great feature and desirable for biomaterial industry.

## Abbreviations

MWCNTs: multi-walled carbon nanotubes; N+-bombarded MWCNTs: N^+^ ion-bombarded multi-walled carbon nanotubes; RBC: red blood cell.

## Competing interests

The authors declare that they have no competing interests.

## Authors' contributions

DL and HG designed this work. MZ, YC, and XL performed the experiments; MZ collected and analyzed the data and wrote the manuscript. JD supported the experiments. All authors read and approved the final manuscript.

## Authors' information

MZ is an Assistant Experimentalist in the College of Physics and Materials Science, Tianjin Normal University, Tianjin, China. YC and XL are Masters degree candidates of College of Physics and Materials Science, Tianjin Normal University, Tianjin, China. JD is a Lecturer in the College of Physics and Materials Science, Tianjin Normal University, Tianjin, China. DL is a Professor in the College of Physics and Materials Science, Tianjin Normal University, Tianjin, China. HG is a Professor in Tianjin Institute of Urological Surgery, Tianjin Medical University, Tianjin and in School of Medicine, Ninth People's Hospital, Shanghai Jiao Tong University, Shanghai, China.

## References

[B1] Liu SB Wei L Hao L Fang N Matthew WC Xu R Yang YH Chen Y Sharper and faster “nano darts” kill more bacteria: a study of antibacterial activity of individually dispersed pristine single-walled carbon nanotube ACS Nano 2009 9 3891 3902 10.1021/nn901252r 19894705

[B2] Kolosnjaj-Tabi J Hartman KB Boudjemaa S Ananta JS Morgant G Szwarc H Wilson LG Moussa F In vivo behavior of large doses of ultrashort and full-length single-walled carbon nanotubes after oral and intraperitoneal administration to Swiss mice ACS Nano 2010 9 1481 1492 10.1021/nn901573w 20175510

[B3] Yan PH Wang JQ Wang L Liu B Lei ZQ Yang SG The in vitro biomineralization and cytocompatibility of polydopamine coated carbon nanotubes Appl Surf Sci 2011 9 4849 4855 10.1016/j.apsusc.2010.12.111

[B4] Magrez A Seo JW Smajda R Mionić M Forró M Catalytic CVD synthesis of carbon nanotubes: towards high yield and low temperature growth Materials 2010 9 4871 4891 10.3390/ma3114871 PMC5445774 28883358

[B5] Li RB Wu RA Zhao L Wu M Yang L Zou H P-glycoprotein antibody functionalized carbon nanotube overcomes the multidrug resistance of human leukemia cells ACS Nano 2010 9 1399 1408 10.1021/nn9011225 20148593

[B6] Dumortier H Lacotte S Pastorin G Marega R Wu W Bonifazi D Briand JP Prato M Muller S Bianco A Functionalized carbon nanotubes are non-cytotoxic and preserve the functionality of primary immune cells Nano Lett 2006 9 1522 1528 10.1021/nl061160x 16834443

[B7] Sayes CM Liang F Hudson JL Mendez J Guo W Beach JM Moore VC Doyle CD West JL Billups WE Ausman KD Colvin VL Functionalization density dependence of single-walled carbon nanotubes cytotoxicity in vitro Toxicol Lett 2006 9 135 142 10.1016/j.toxlet.2005.08.011 16229976

[B8] Yen SJ Hsu WL Chen YC Su HC Chang YC Chen H Yeh SR Yew TR The enhancement of neural growth by amino-functionalization on carbon nanotubes as a neural electrode Biosens Bioelectron 2011 9 4124 4132 10.1016/j.bios.2011.04.003 21536420

[B9] Coccini T Roda E Sarigiannis DA Mustarelli P Quartarone E Profumo A Manzo L Effects of water-soluble functionalized multi-walled carbon nanotubes examined by different cytotoxicity methods in human astrocyte D384 and lung A549 cells Toxicology 2010 9 41 53 20079395 10.1016/j.tox.2010.01.005

[B10] Zhao ML Li DJ Yuan L Liu H Sun X Differences in cytocompatibility and hemocompatibility between carbon nanotubes and nitrogen-doped carbon nanotubes Carbon 2011 9 3125 3133 10.1016/j.carbon.2011.03.037

[B11] Zhang YT Li DJ Zhao ML Guo MX Deng XY Gu HQ Wan RX Differences in cytocompatibility between MWCNTs and carboxylic functionalized MWCNTs Funct Mater Lett 2013 9 1250053 10.1142/S1793604712500531

[B12] Zhang YT Li DJ Zhao ML Guo MX Deng XY Sun X Geng D Gu HQ Comparison in cell and blood behaviors of pristine, carboxyl and hydroxyl functionalized multiwalled carbon nanotubes Sci Adv Mater 2013 9 1436 1443 10.1166/sam.2013.1640

[B13] Guo MX Li DJ Zhao ML Zhang YT Geng D Li R Sun X NH_2_^+^ implantations induced superior hemocompatibility of carbon nanotubes Nanoscale Res Lett 2013 9 205 208 10.1186/1556-276X-8-205 23634977 PMC3660171

[B14] Zhang YT Li MS Zhao ML Li DJ Influence of polar functional groups introduced by COOH^+^ implantation on cell growth and anticoagulation of MWCNTs J Mater Chem B 2013 9 5543 5549 10.1039/c3tb21011a 32261178

[B15] Guo MX Li DJ Zhao ML Zhang YT Geng D Lushington A Sun X Nitrogen ion implanted graphene as thrombo-protective safer and cytoprotective alternative for biomedical applications Carbon 2013 9 321 328

[B16] Guo MX Li MS Liu XQ Zhao ML Li DJ Geng D Sun X Gu HQ N-containing functional groups induced superior cytocompatible and hemocompatible graphene by NH_2_ ion implantation J Mater Sci Mater Med 2013 9 2741 2748 10.1007/s10856-013-5016-0 23907737

[B17] Zhao ML Li DJ Guo MX Zhang YT Gu HQ Deng XY Wan RX Sun X The different N concentrations induced cell and blood compatibility of MWCNTs with CN_x_ coatings Surf Coat Technol 2013 9 90 96

[B18] Zhao ML Li DJ Gu HQ Guo MX Zhang YT In vitro cell adhesion and hemocompatibility of carbon nanotubes with CN_x_ coating Curr Nanosci 2012 9 451 457 10.2174/157341312800620359

[B19] Li DJ Yuan L Yang Y Deng XY Lü XY Huang Y Cao Z Liu H Sun X Adsorption and adhesion of blood protein and fibroblast on multi-wall carbon nanotubes Sci China C Life Sci 2009 9 479 482 10.1007/s11427-009-0049-9 19471872

[B20] Carrero-Sánchez JC Elίas AL Mancilla R Arrellín G Terrones H Laclette JP Terrones M Biocompatibility and toxicological studies of carbon nanotubes doped with nitrogen Nano Lett 2006 9 1609 1616 10.1021/nl060548p 16895344

[B21] Shirasaki T Moguet F Lozano L Tressaud A Nanse G Papire E Fluorination of carbon blacks: an X-ray photoelectron spectroscopy study: IV. Reactivity of different carbon blacks in CF_4_ radiofrequency plasma Carbon 1999 9 1891 1900 10.1016/S0008-6223(99)00066-4

[B22] Nansé G Papirer E Fioux P Moguet F Tressaud A Fluorination of carbon blacks: an X-ray photoelectron spectroscopy study: III. Fluorination of different carbon blacks with gaseous fluorine at temperatures below 100°C influence of the morphology, structure and physico-chemical characteristics of the carbon black on the fluorine fixation Carbon 1997 9 515 528 10.1016/S0008-6223(97)00003-1

[B23] Tabbal M Merel P Moisa S Chaker M Ricard A Moisan M X-ray photoelectron spectroscopy of carbon nitride films deposited by graphite laser ablation in a nitrogen postdischarge Appl Phys Lett 1996 9 1698 1700 10.1063/1.118000

[B24] Xu P Li JJ Wang Q Gu CZ Cui Z Improving mechanical properties of amorphous carbon nitride films by titanium doping J Appl Phys 2007 9 14312 14316 10.1063/1.2404797

[B25] Liu H Zhang Y Li RY Sun XL Désilets S Abou-Rachid H Jaidann M Lussier LS Structural and morphological control of aligned nitrogen-doped carbon nanotubes Carbon 2010 9 1498 1507 10.1016/j.carbon.2009.12.045

[B26] Chen YG Wang JJ Meng XB Zhong Y Li RY Sun XL Ye SY Knights S Atomic layer deposition assisted Pt-SnO_2_ hybrid catalysts on nitrogen-doped CNTs with enhanced electrocatalytic activities for low temperature fuel cells Int J Hydrogen Energy 2011 9 11085 11092 10.1016/j.ijhydene.2011.05.156

[B27] Deepak FL John NS Govindaraj A Kulkarni GU Rao CNR Nature and electronic properties of Y-junctions in CNTs and N-doped CNTs obtained by the pyrolysis of organometallic precursors Chem Phys Lett 2005 9 468 473

[B28] Charpentier PA Maguire A Wan WK Surface modification of polyester to produce a bacterial cellulose-based vascular prosthetic device Appl Surf Sci 2006 9 6360 6367 10.1016/j.apsusc.2005.09.064

[B29] Namgung S Baik KY Park J Hong S Controlling the growth and differentiation of human mesenchymal stem cells by the arrangement of individual carbon nanotubes ACS Nano 2011 9 7383 7390 10.1021/nn2023057 21819114

[B30] Yang W Cui FZ Qing XL Behavior of phosphatidylcholine adsorption on CN_x_ coated PTFE films Curr Appl Phys 2006 9 827 832 10.1016/j.cap.2005.01.033

[B31] Ferrari AC Rodil SE Robertson J Interpretation of infrared and Raman spectra of amorphous carbon nitrides Phys Rev Biol 2003 9 155306 155325

[B32] Horbett TA The role of adsorbed proteins in animal cell adhesion Colloids Surf B Biointerfaces 1994 9 225 240 10.1016/0927-7765(94)80037-5

[B33] Takemoto S Kusudo Y Tsuru K Hayakawa S Osaka A Takashima S Selective protein adsorption and blood compatibility of hydroxy-carbonate apatites J Biomed Mater Res 2004 9 544 551 10.1002/jbm.a.30039 15127401

[B34] Yokota T Terai T Kobayashi T Iwaki M Cell adhesion to nitrogen-doped DLCS fabricated by plasma-based ion implantation and deposition method Nucl Instrum Methods Phys Res B 2006 9 48 50 10.1016/j.nimb.2005.08.107

[B35] Lacerda SHDP Semberova J Holada K Simakova O Hudson SD Simak J Carbon nanotubes activate store-operated calcium entry in human blood platelets ACS Nano 2011 9 5808 5813 10.1021/nn2015369 21639133

[B36] Baurschmidt P Schaldach M Alloplastic materials for heart-valve prostheses Med Biol Eng Comput 1980 9 496 502 10.1007/BF02443327 7421344

[B37] Owens AP Mackman N Tissue factor and thrombosis: the clot starts here Thromb Haemost 2010 9 432 439 10.1160/TH09-11-0771 20539911 PMC3043984

[B38] Zhang L Chen M Li ZY Chen DH Pan SR Effect of annealing on structure and haemocompatibility of tetrahedral amorphous hydrogenated carbon films Mater Lett 2008 9 1040 1043 10.1016/j.matlet.2007.07.042

[B39] Gao JC Li LC Wang Y Qiao LY Corrosion resistance of alkali heat treated magnesium in bionics simulated body fluid Rare Metal Mater Eng 2005 9 903 907

[B40] Alanazil AS Hirakuri KJ Blood compatibility of DLC films Eur Cells Mater 2010 9 15 20

